# Collodion baby: A rare case report

**DOI:** 10.1016/j.ijscr.2023.108930

**Published:** 2023-10-11

**Authors:** Maryem Bouab, Oumaima Wajih, Asmaa Assal, Mohamed Jalal, Amine Lamrissi, Said Bouhya

**Affiliations:** Department of gynecology and obstetrics, University hospital center Ibn Rochd, Casablanca, Morocco; Faculty of Medicine and Pharmacy, Hassan II University, Casablanca, Morocco

**Keywords:** New-born, Collodion baby, Dermatological affection-premature delivery

## Abstract

**Introduction:**

Collodion baby “CB” is an extremely rare dermatological condition.

Approximately 1 in 100,000 births are identified as infants with CB syndrome, including stillbirths (Dyer et al., 2013).

A cornified substance replaces the newborn's skin, giving the body a varnished or parchment-like appearance.

**Case presentation:**

Patient aged 30 years, third gesture, third pare, admitted for premature delivery of 8 months. After labor management, she gave birth 2 h after admission to the maternity ward of a living newborn female weighing 2400 g. The initial physical examination revealed large, thick scales all over the body. Examination of the head and neck revealed an abnormal parchment-like membrane covering the head and sparse hairs.

Excessive scaling around the mouth gives a typical fish-like appearance. No other obvious abnormalities were observed.

**Clinical discussion:**

CB is an extremely rare dermatological condition. This is a disorder secondary to cornification. These children are generally born prematurely, and are not diagnosed until after birth.

Due to the presence of a tight membrane, these babies develop numerous complications such as eclabium, ectropion, limited movement of the extremities and fingers. Treatment consists mainly of support, such as the use of intravenous fluids, incubators, tube feeding and emollients.

**Conclusion:**

The collodion baby is a newborn characterized by an altered skin barrier, exposing him or her to numerous complications. Fortunately, the mortality rate has fallen thanks to improved neonatal care.

## Highlights

• Collodion baby is a rare congenital disorder with life-threatening complications.
• A cornified substance replaces the newborn's skin, giving the body a varnished or parchment-like appearance
• Early diagnosis is important to minimize the physiological and psychological impact of such anomalies on the mother and family.

## 1 Introduction

Collodion baby “CB” is an extremely rare dermatological condition.

Approximately 1 in 100,000 births are identified as infants with CB syndrome, including stillbirths [3].

A cornified substance replaces the newborn's skin, giving the body a varnished or parchment-like appearance [2].

We report this rare case to create awareness among people about the early diagnosis of this dermatological anomaly.

This work has been reported with respect to the SCARE 2020 criteria [8].

## 2 Case presentation

Patient aged 30 years, third gesture third pare, mother of two children alive by vaginal route was admitted in the labor room of the Ibn Rochd university hospital center of Casablanca for a premature delivery on badly followed pregnancy presumed at 8 months. On questioning the parturient, there was no notion of consanguinity with her spouse. The parents did not mention the history of congenital anomalies or close family marriage in their relative. There was no notion of taking medication or plants during the pregnancy. There was no history of dermatological disorders or other genetic diseases. The fasting blood sugar level was within normal limits during the pregnancy. No obstetrical ultrasound was performed during her pregnancy.

On admission, the patient was conscious, the blood pressure was 11/6cmHg, the urine dipstick did not show any proteinuria. Her cervix was dilated to 6 cm with intact membranes.

On obstetrical ultrasound, we found a progressive monofetal pregnancy, with positive cardiac activity, ultrasound biometrics corresponded to the term. There was an associated hydramnios with a large cistern that was 11 cm, the placenta was fundial.

After labor management, she gave birth 2 h after admission to the maternity ward of a living newborn female weighing 2400 g. The newborn's Apgar score apgar score was 8.

The initial physical examination revealed large, thick scales all over the body. Examination of the head and neck revealed an abnormal parchment-like membrane covering the head and sparse hairs.

**Fig. 1:**
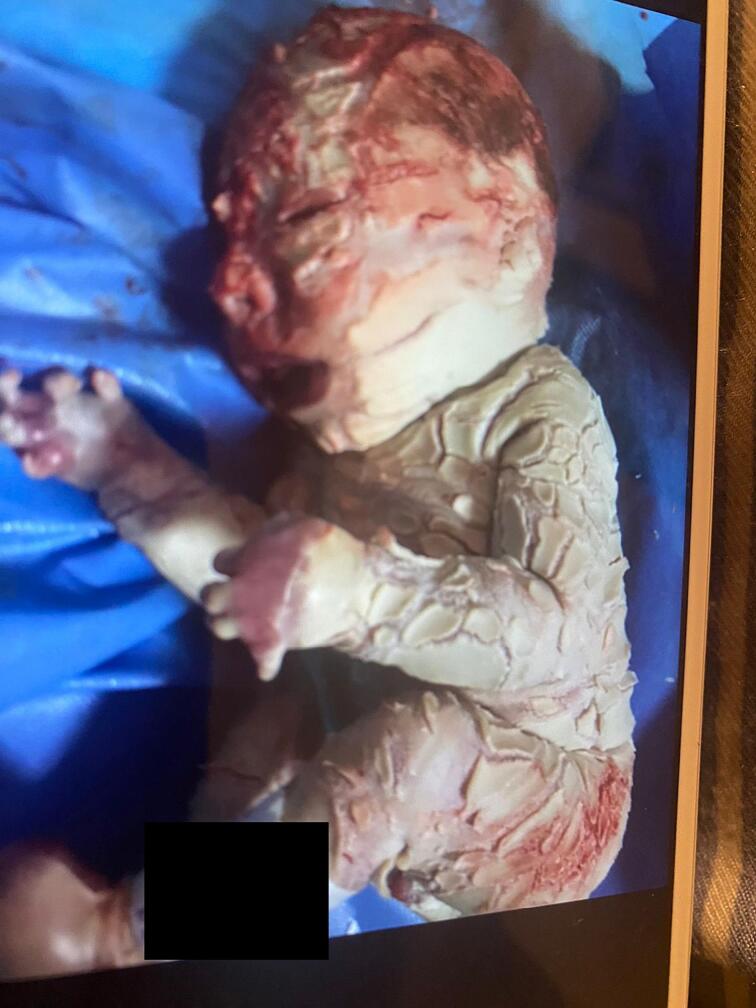
Image of the newborn.

**Fig. 2:**
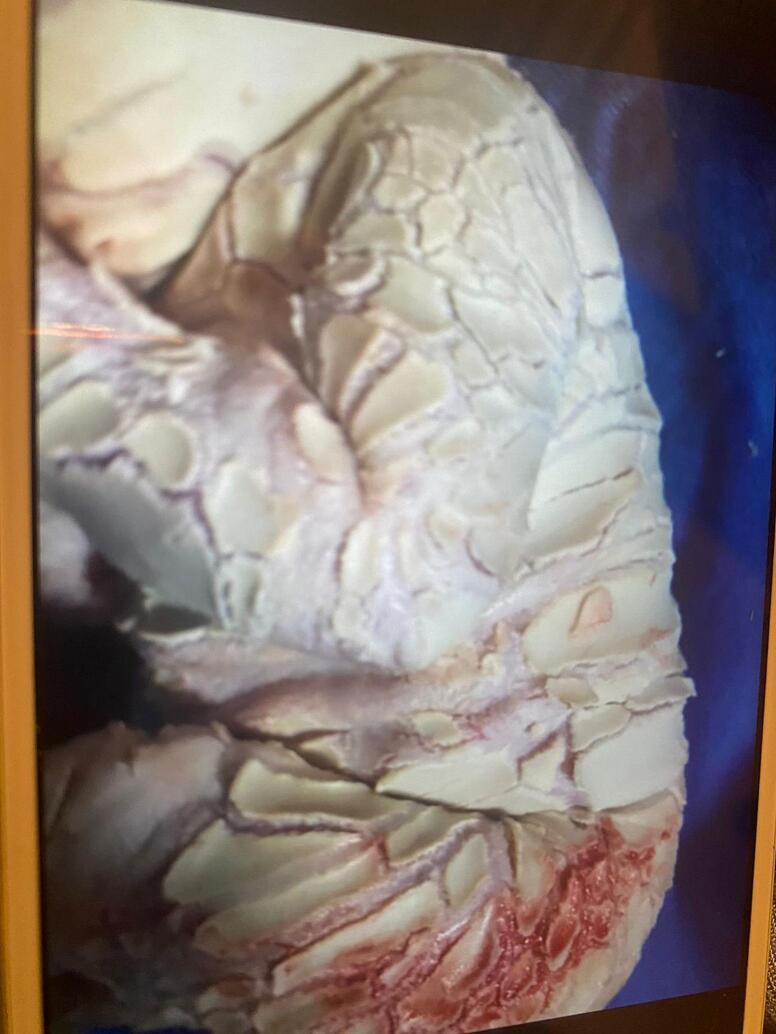
image showing cornification defect.

Excessive scaling around the mouth gives a typical fish-like appearance. No other obvious abnormalities were observed. A thorough systemic examination was carried out and revealed no abnormalities (Fig. 1, Fig. 2).

A cord sample was taken, the parents refused to do a karyotype.

The newborn was admitted to the neonatal intensive care unit and managed with adequate humidification in an incubator. He expired within 10 h after birth. Unfortunately, her parents did not give consent for an autopsy.

## 3 Discussion

This case report we presented is a unique case with severe congenital icthyosis diagnosed in the postnatal period in a neonate born in Ibn Rochd university hospital center of Casablanca in Morocco.

Watelet and Hallopeau were the first to use the term “collodion baby” (CB) [1].

A cornified substance replaces the newborn's skin, giving the body a varnished or parchment-like appearance [2].

CB is an extremely rare dermatological condition, its incidence is estimated to 1 in 50,000 to 100,000 birth [1].

This is a disorder secondary to cornification. These children are generally born prematurely, and are not diagnosed until after birth.

Due to the presence of a tight membrane, these babies develop numerous complications such as eclabium, ectropion, limited movement of the extremities and fingers, absence of eyebrows, sparse hair on the head, deformed nose and ears due to hypoplasia of the nasal and auricular cartilage. These newborns have poor sucking, ischemia of the distal limbs and edema of the extremities [4].

Detachment of the collodion membrane is observed within 4 weeks of birth, revealing the underlying skin disorder. In the long term, around 75 % of collodion babies will develop congenital ichthyosis (congenital ichthyosiform erythroderma or lamellar ichthyosis) [5].

The exact etiology of CB syndrome is not well elucidated, but in most cases there is an autosomal recessive mode of inheritance, which is very rare and may be associated with consanguinity [6].

Collodion syndrome is rare, which is why it's important to have a protocol for treating these patients, with instructions for treatment and proper management of any complications that may arise.

Treatment consists mainly of support, such as the use of intravenous fluids, incubators, tube feeding and emollients. Particular attention must be paid to the skin, and the collodion membrane must not be removed, as it will fall off after one to two weeks. Infections must also be avoided [7].

In the case of long-term treatment, the cause of the illness must be established so that the necessary measures can be taken for the patient.

## 4 Conclusion

In conclusion, the collodion baby is a newborn characterized by an altered skin barrier, exposing him or her to numerous complications including hypernatremic dehydration, infection and hypothermia. Fortunately, the mortality rate has fallen thanks to improved neonatal care.

Collodion babies should be placed in a humidified incubator and closely monitored for the first few weeks of life.

## Patient consent

Written informed consent was obtained from the patient's family for publication of this case report. A copy of the written consent is available for review by the Editor-in-Chief of this journal on request.

## Ethical approval

The case report was ethically approved by the ethical committee of our university Hassan 2 of Casablanca.

## Funding

None.

## Author contribution

Bouab Maryem: Corresponding author and writing the paper.

Wajih Oumaima: writing the paper.

Assal Asmaa: study concept.

Jalal Mohamed: study concept.

Lamrissi Amine: study concept.

Bouhya Said: correction of the paper.

All authors have read and approved the final version of the manuscript.

## Guarantor

DR Bouab Maryem.

## Research registration number

None.

## Conflict of interest statement

The authors declare having no conflicts of interest for this article.
